# Increased Expression of Toll-Like Receptor 4 in Skin of Dogs with Discoid Lupus Erythematous (DLE)

**DOI:** 10.3390/ani11041044

**Published:** 2021-04-08

**Authors:** Alessandro Di Cerbo, Sara Giusti, Francesca Mariotti, Andrea Spaterna, Giacomo Rossi, Gian Enrico Magi

**Affiliations:** School of Biosciences and Veterinary Medicine, University of Camerino, Via Circonvallazione 93/95, 62024 Matelica, Italy; sara.giusti@studenti.unicam.it (S.G.); francesca.mariotti@unicam.it (F.M.); andrea.spaterna@unicam.it (A.S.); giacomo.rossi@unicam.it (G.R.); gianenrico.magi@unicam.it (G.E.M.)

**Keywords:** dog, DLE, TLR4, skin, Toll-like receptors

## Abstract

**Simple Summary:**

Discoid lupus erythematous (DLE) is a common autoimmune skin disorder of dogs. The aim of this study was to evaluate the expression of TLR4, a bacterial lipopolysaccharide sensor, in skin of dogs with DLE and in normal skin to evaluate a possible involvement of this receptor in the disease pathogenesis. Skin samples of affected dogs had a diffuse and intense expression of TLR4, both in the epidermis and in inflammatory infiltrates. The expression was higher in DLE skin compared to normal skin. The high TLR4 expression might play an important pathogenic role in the ongoing immunopathologic process.

**Abstract:**

Discoid lupus erythematous (DLE) is a common autoimmune skin disorder of dogs where keratinocytes play a pivotal role both in the innate and adaptive immune responses. As for the innate response, pattern recognition receptors (PRR), including Toll-like receptors (TLRs), can activate macrophages and immune tissue cells allowing for transmission and transduction of signals through cytokine and chemokine release to improve host defenses. In particular, TLR4 can also recognize endogenous molecules such as heat shock proteins produced during reactions to tissue damage. The aim of this study was to evaluate the expression of TLR4, a bacterial lipopolysaccharide sensor, in the skin of dogs with DLE and in normal skin to evaluate a possible involvement of this receptor in the disease pathogenesis. Skin samples of affected dogs had a diffuse and intense expression of TLR4 in the epidermis. Also, the inflammatory infiltrates were immunolabelled. The expression was significantly higher in DLE skin compared to normal skin (**** *p* < 0.0001). In conclusion, dogs with DLE showed an altered expression of TLR4, which might play an important pathogenic role in the ongoing immunopathologic process, thus being considered a valuable therapeutic potential target for DLE.

## 1. Introduction

Dermatological issues represent almost 25% of cases referred to veterinary clinics [1]. In this sense, keratinocytes play a pivotal role both in innate and adaptive immune responses [2]. As for the innate response, it is possible to observe the activation of macrophages and immune tissue cells through pattern recognition receptors (PRR), able to distinguish between pathogen-associated molecular patterns (PAMP) and damage-associated molecular patterns (DAMP) [3]. Cell PRRs are represented by Toll-like receptors (TLRs), NOD-like receptors (NRL), RIG-like receptors (RLR), cytosolic DNA sensors (CDS), scavenger receptors, and other soluble proteins such as *pentraxins, collectins,* *ficolins, and the complement system* [4]. TLRs allow transmission and transduction of signals through cytokine and chemokine release to improve host defenses [2]. Thus, TLR activation patterns play key roles in the interaction between innate and adaptive immune responses. In particular, TLR4, a receptor known to bind bacterial LPS, can also recognize endogenous molecules e.g., heat shock proteins, type III fibronectin, and saturated fatty acids produced during reactions to tissue damages [5].

PRR activation is, therefore, essential to trigger cutaneous defense mechanisms against invasive pathogens, but its hyperactivation might result into uncontrolled inflammatory diseases and/or cutaneous autoimmune diseases [4]. Canine autoimmune dermatopathies represent 1–2% of cutaneous pathologies, with pemphigus and lupus erythematosus (LE) being the most common, particularly in some more predisposed breeds [6]. Moreover, despite the unclear etiology of LE, it has been argued that ulcerative and localized cutaneous lesions might be worsened by sunlight [2]. According to Gilliam and Sontheimer, there are two different canine cutaneous LE subsets, LE-specific (Vesicular, espholiative, discoid, or mucocutaneous) and LE non-specific cutaneous diseases [7].

As for discoid lupus erythematosus (DLE), it is the most common form both in human and animal species, being either localized to the facial region (FDLE) [8] or generalized, extending beyond the neck (GDLE) [9,10]. As far as the localized form is concerned, the age of onset of the lesions is around seven years regardless of sex, with a predisposition in German Shepperd dogs and their crosses [10]. The first cutaneous lesions of FDLE consist of erythema, depigmentation, and desquamation that progress in discoid erosions, ulcerations and atrophy, and possibly pruritus [10]. Conversely, dogs with GDLE show discoidal, multifocal, or generalized plaques with pigmentation alterations, erythematosus margins, and central alopecia in the neck, back, and thoracic region. In most of these dogs, plaques evolve into scar ulcerations characterized by an atrophic or hypertrophic central scar and an altered pigmentation [9].

Histologically, DLE is characterized by a typical interfacial pro-inflammatory pattern, rich in lymphocytes, with a vacuolar degeneration and/or apoptosis of basal keratinocytes, along with a slight thickening of the basal membrane, which is also present in the course of leishmaniasis [11]. On the contrary, GDLE interfacial dermatitis develops in the lesion boundaries. The epidermis can be atrophic or slightly hyperplastic, and depigmentation pronounced at boundaries. In chronic lesions, dermal fibrosis can confine the inflammatory infiltrate to the superficial dermis. Despite limited data about pharmacological treatment of FDLE, topical application of tacrolimus 0.1% with or without doxycycline/niacinamide combined with essential fatty acids and vitamin E seems to be the best option [12].

Notwithstanding the good response of GDLE to a wide number of treatments, most dogs have a relapse after gradual dosage decrease [9]. Nevertheless, a significant improvement or a complete remission was observed after oral administration of ciclosporin (4.8 mg/kg/d) along with a short course of glucocorticoids [9].

The aim of this study was to evaluate the immunohistochemical expression of toll-like receptor 4 in DLE and normal skin of dogs to shed light on a possible involvement of this receptor in the disease physio-pathogenesis.

## 2. Materials and Methods

Twenty-four *post-mortem* samples, 8 from planum nasale of healthy dogs and 16 from planum nasale and/or dorsal muzzle of dogs with DLE, confirmed by histopathologic diagnosis, were collected from 2012 to 2019 at the Pathology Unit of the School of Bioscience and Veterinary Medicine of the University of Camerino (Italy) and included in this study. The specimens were fixed in 10% neutral buffered formalin, subsequently processed by an automatic histoprocessor, and embedded in paraffin. Serial sections (3 µm) were stained with hematoxylin and eosin (HE).

Pathological cases were referrable to different breeds with age ranging from 2 to 10 years, 62% male and 38% female. A serological ELISA test (Leiscan, Esteve) for leishmaniasis was carried out in all of the 16 cases, which resulted negative. In addition, immunohistochemical analysis with a monoclonal antibody anti-Kinetoplast Membrane Protein-11 (KPM11) confirmed the lack of *Leishmania* organisms.

All the cases resulted negative also for anti-nuclear antibody (ANA) test. Clinical-anamnestic data of all dogs were collected and summarized in Table 1.

**Figure 1:**
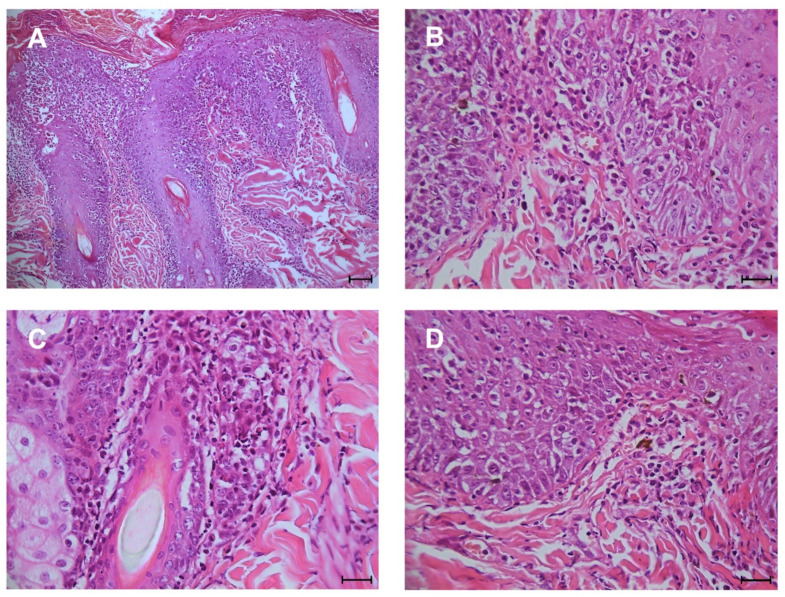
Histopathologic features of DLE. (**A**) interface dermatitis (bar 75 µm); (**B**,**C**) infiltrate of plasma cells and lymphocytes within dermo-epidermal junction and hydropic degeneration of keratinocytes of stratum basale (scale bar 25 µm); (**D**) scattered macrophages contain melanin (pigmentary incontinence) within the superficial dermis (scale bar 25 µm).

Typical microscopic findings of DLE, considered as diagnostic criteria for including the cases in this study, consist in: interface (lichenoid) dermatitis characterized by a band of inflammatory cells, predominantly lymphocytes at the dermo-epidermal junction; hydropic degeneration and apoptosis (Civatte bodies) of the epidermal basal cells; melanin-laden macrophages (pigmentary incontinence) within superficial dermis (Figure 1).

### 2.1. Immunohistochemical Analysis

Immunohistochemical analysis was carried out with a standard ABC-peroxidase method on sections with a thickness of 3 µm as described by Mariotti et al. (2019) [13]. Sections were pre-treated with citrate buffer for antigen-retrieval, and the labelling expression of TLR4 was evaluated using a mouse monoclonal antibody anti-TLR4, clone 76B357.1, developed against a portion of 100–200 amino acids of human TLR4 (NP_612564, Novus Biologicals, Milan, Italy) diluted 1:100. As secondary antibody, a Goat anti-mouse biotinylated antibody was used along with DAB (diaminobenzidine) as chromogen. Sections were counterstained in Mayer’s haematoxylin. A blocking peptide-based protocol for TLR4 was used as negative control (Supplementary Material). Labelling was evaluated by two pathologists (semi-quantitatively based on the percentage of immunopositive cells and staining intensity to obtain an expression score. An optical microscope (Leica DM 2500, Leica Microsystems Srl, Buccinasco, Italy) equipped with a camera (Leica DFC 7000T, Leica Microsystems Srl, Buccinasco, Italy) was used to acquire pictures. Each sample was firstly observed at 10× and then 5 microscopic fields at 40× were chosen for the semiquantitative evaluation. A score was then assigned to each sample according to positive cells percentage and their signal intensity.

Positive cells percentage was based on a 5-point scoring system: 0 = none positive cell, 1 = positive cells ranging from 0 to 25%, 2 = positive cells ranging from 26 to 50%, 3 = positive cells ranging from 51 to 75%, 4 = positive cells > 75%. For each microscopic field, a count of immunopositive and negative cells was carried out and then transformed in percentage.

Regarding the intensity of the immunolabeled, it was evaluated according to a 4-point scoring system: 0 = no staining, 1 = low-intensity staining, 2 = moderate-intensity staining, 3 = high-intensity staining. In those samples where intensity was heterogeneous the score chosen was the predominant one in the sample itself. The overall score assigned to each sample derived by the multiplication of cell positivity and intensity signal score, with a minimum score of 0 and a maximum score of 12. The equivocal cases were evaluated together by two authors (G.E.M. and F.M.) to establish the score.

### 2.2. Statistical Analysis Mann-Whitney

Data were analyzed using GraphPad Prism8 software (GraphPad Software, Inc., La Jolla, CA, USA). Difference in intensity score between DLE and normal skin cases was analyzed by a Mann-Whitney test. A * *p* < 0.05 was considered significant.

## 3. Results

**Figure 2:**
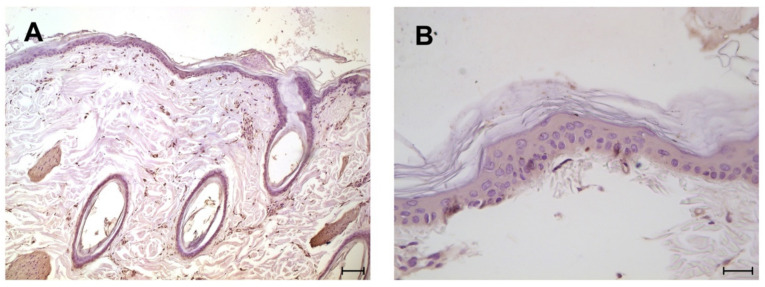
Immunohistochemical analysis for TLR4: normal epidermis with a low and diffuse intensity expression; (**A**) scale bar 75 µm; (**B**) scale bar 25 µm.

TLR4 monoclonal antibody demonstrated its suitability for normal and DLE skin fixed in neutral buffered formalin, the pre-treatment of the sections with the blocking peptide specific for the antibody used ruled out any possible false positive reactivity. The 8 samples of normal skin always showed a diffuse, positive reactivity ranging from mild (6 cases) to moderate (2 case) in the cytoplasm of epithelial cells of the epidermal layer, including those of the hair infundibulum and annexes (Figure 2). Moreover, an expression of the muscle cells of the erector pilar muscle was observed.

**Figure 3:**
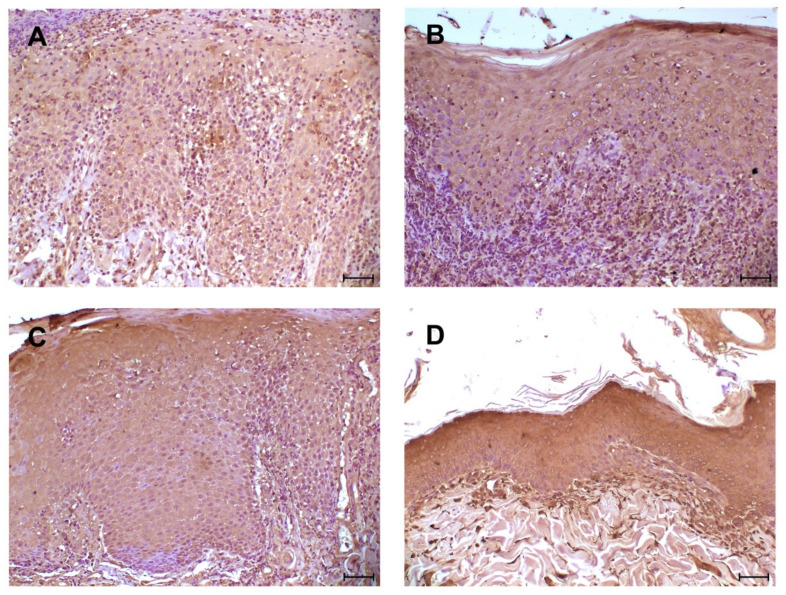
Immunohistochemical analysis for TLR4: four different cases of DLE with epidermis diffusely positive; (**A**) moderate intensity staining of the immunolabeled; (**B**–**D**) high-intensity staining of inflammatory cells. Diffuse immunolabelling of inflammatory cells at dermo-epidermal junction. (**A**–**D**) scale bar 50 µm.

As for the 16 cases of DLE, a diffuse, positive reactivity of keratinocytes of the epidermal layer was observed with a labeling intensity ranging from moderate (3 cases) to intense (13 cases). Epidermal epithelial cells showed a cytoplasmic immunopositivity with membrane reinforcement (Figure 3). All DLE cases showed a diffuse immunopositivity of inflammatory cells (in particular macrophages and lymphocytes).

The means of scores of DLE and normal skin cases are reported in Table 2. It is possible to observe a significant increase in DLE score with respect to that of the normal skin, 11.25 ± 1.6 and 4.5 ± 1, respectively (**** *p* < 0.0001), despite observing a positivity.

## 4. Discussion

Recent human studies demonstrated that TLR4 plays a pivotal role in autoimmune diseases, including DLE [14,15], and therefore might represent a potential target for future immunotherapeutic approaches. Beyond the binding of the lipopolysaccharide, TLR4 can also recognize both exogenous (viral glycoproteins, respiratory syncytial virus fusion proteins, and murine mammary cancer virus envelope protein) and endogenous (necrotic cells, HSP, fibronectin, fatty acids, low-density lipoproteins, and fibrinogen) ligands [14,16].

The TLR4 expression analysis carried out in normal and DLE skin sections of dogs allowed us to observe its low expression in the former and a higher expression in the latter. Intriguingly, the low TLR4 expression in normal skin seems to be in contrast with literature reports, which did not report any expression in such tissue [17]. The results might have a bias since the normal skin samples used in this study were obtained by *post-mortem* cases and, therefore, possible changes between death and fixation might have occurred, as reported in other studies [18,19]. However, it is undeniable that the death of the organism causes an initial peripheral hypoxia that worsens by the hour, and that the single cells, which obviously do not die with the individual, undergo a stressful event producing intrinsic signals. This could modulate the expression levels of TLR4, but also of other ligands, and therefore if the use of *post-mortem* samples is ethically accepted *postmortem* studies could present some limitations, and the results should always be interpreted considering this aspect.

In our study, six normal skin cases showed weak intensity of the immunolabeled, while only two showed a moderate intensity. We cannot rule out that this latter result might reflect a possible slight exposure to some environmental ligand/agent.

In human medicine, TLR4 expression in normal tissues is quite controversial, in particular at the epidermis level [20,21,22]. In this study, the localization of the immunolabeled in the keratinocytes of normal and DLE skin was mainly cytoplasmic, as reported by human studies [21,22,23]. In addition, an increase in TLR4 cutaneous expression was also observed in human pemphigus and systemic lupus erythematosus [15,22].

Likewise, a high TLR4 expression in the epidermis and inflammatory cells (lymphocytes and histiocytes) in biopsies of dogs with DLE are also reported. This marked expression might indicate a functional role of TLR4 during a chronic inflammatory cutaneous condition such as DLE.

However, in human medicine, the role of TLR4 in pro-inflammatory cytokine production and inflammatory cells recruitment is widely documented [24]. The high TLR4 expression in skin lesions of dogs with DLE might be linked to the presence and/or high levels of endogenous ligands, e.g., HSPs. Human studies demonstrated that intracellular HSPs synthesis increases during inflammatory and autoimmune diseases [16].

Therefore, HSPs-induced activation of TLR4 during DLE, followed by several pro-inflammatory cytokine production, might worsen this chronic inflammatory condition through a positive-feedback mechanism. It has been also demonstrated that hsp70 induces pro-inflammatory cytokine production through MyD88/NFκB pathway and that uses both TLR2 (Gram-positive receptor) and TLR4, by means of CD14, to transduce its pro-inflammatory signal [25].

In recent years, TLR4 has been directly implicated in the mechanisms and regulation of necroptosis, a newly discovered pathway of regulated necrosis induced by several stimuli, including toll-like receptors, through activation of the necrosome [26,27].

Degeneration and necrosis characteristically observed in the epidermal basal layer during DLE with associated interface inflammation could be therefore induced by such regulated cell death. In support of this hypothesis, recently in human medicine, it has been demonstrated that both necroptosis and the RIP3-dependent NLRP3 inflammasome pathways are activated in podocytes during lupus nephritis [28], likewise a type I immunity has been associated with keratinocyte necroptosis in lichen planus and lupus erythematosus, two forms of interface dermatitis [29].

## 5. Conclusions

In conclusion, we can affirm that dogs with DLE show an altered expression of TLR4, although it still remains unclear how such alteration can functionally affect the disease onset and/or progression. The high TLR4 expression might play an important pathogenic role in the ongoing immunopathogenic process. Thus, TLR4 might be considered a valuable therapeutic potential target for DLE.

## Figures and Tables

**Table 1:** Schematic representation of the 16 cases of discoid lupus erythematous (DLE) collected during the study (N = negative).

Case N°	Sex	Age (Years)	Breed	*Leishmania* Elisa-Test	*Ana*-Test
1	Female	5	German Shepperd	N	N
2	Male	10	Cocker	N	N
3	Male	2	Akita Inu	N	N
4	Male	2	Maremma Shepperd	N	N
5	Male	7	Crossbred	N	N
6	Female	4	Collie	N	N
7	Male	3	Collie	N	N
8	Female	4	Retriever	N	N
9	Female	4	Crossbred	N	N
10	Female	2	American Akita	N	N
11	Male	3	German Shepperd	N	N
12	Male	2	Labrador	N	N
13	Female	6	Crossbred	N	N
14	Male	5	Pinscher	N	N
15	Male	5	German Shepperd	N	N
16	Male	7	Golden Retriever	N	N

**Table 2:** Mean (±SD) score of TRL4 expression in DLE and normal skin; **** *p* < 0.001.

	Mean Score	Minimum Score	Maximum Score	SD	% of Positive Cases
DLE (n = 16)	11.25 ****	8	12	1.6	100 (16/16)
Normal skin (n = 8)	4.5	4	6	1	100 (8/8)

## Data Availability

The data presented in this study are available on request from the corresponding authors.

## Supplementary Materials

The following are available online at https://www.mdpi.com/article/10.3390/ani11041044/s1, Immunohistochemical analysis for TLR4. Case of DLE: negative control using a blocking peptide specific for the TLR4 antibody used; scale bar 50 µm.
