# Supplementary material for: Increased Expression of Toll-Like Receptor 4 in Skin of Dogs with Discoid Lupus Erythematous (DLE)
Source: Animals (Basel). 2021 Apr 8;11(4):1044. doi: 10.3390/ani11041044 (PMC8067959; doi:10.3390/ani11041044)
Supplement: Supplementary file 1 [file animals-11-01044-s001.pdf]

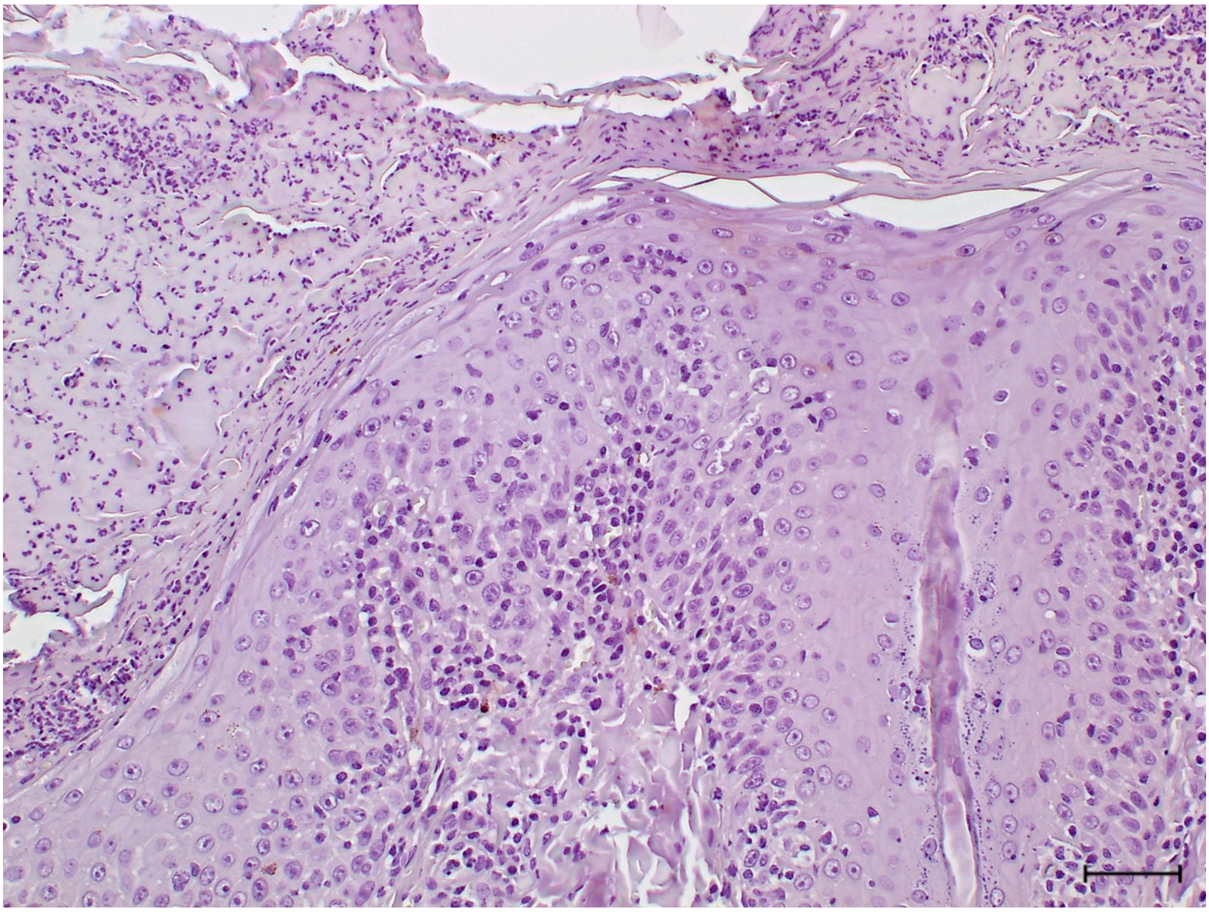

Figure S1. Immunohistochemical analysis for TLR4. Case of DLE: negative control using a blocking peptide specific for the TLR4 antibody used; scale bar 50 μm.
